# Dysuria and Vaginal Pain, Unusual Manifestations of B-acute Lymphoblastic Lymphoma

**DOI:** 10.1177/2632010X241272377

**Published:** 2024-08-16

**Authors:** Ananya Datta Mitra, Anupam Mitra, Jong H Chung, Elham Vali Betts

**Affiliations:** 1 Department of Pathology and Laboratory Medicine, University of California Davis, Sacramento, CA, USA; 2 Department of Pediatrics, Division Pediatric Hematology/Oncology. University of California Davis, Sacramento, CA, USA

**Keywords:** Case report, B-lymphoblastic leukemia, dysuria, vaginal pain, B-lymphoblastic lymphoma

## Abstract

Urinary symptoms are one of the most common reasons for emergency visits in females of pediatric age group and can be associated with various conditions like infections (most common), sexual trauma and rarely neoplastic processes. Here, we report a case of a 7-year-old female who presented in the emergency multiple times with the complaints of urinary symptoms and vaginal pain and was empirically treated with antibiotics and antifungals without symptomatic improvement. Her blood tests, physical examination during this time remained unrevealing. She was then transferred to our institution on her third emergency visit for further evaluation. On imaging studies, she was noted to have expansile lesions on her vertebral body at the L4 and T6 levels with compressive myelopathy with multiple bone and soft tissue lesions throughout her lower extremities. Patient developed saddle anesthesia requiring emergent decompression and biopsy of the epidural mass with the final pathology coming back as B-lymphoblastic leukemia/lymphoma. B-ALL/B-LBL is the most common pediatric hematologic malignancy and usually presents with fever, hepatosplenomegaly, lymphadenopathy, bone pain and bleeding. Occasionally, atypical presentations like bone and joint pain, osteoporosis, palpable paravertebral mass have been described. However, this is the first case report to describe a very unusual and unfamiliar presentation of this disease causing significant diagnostic difficulty resulting in delayed treatment. This case report can aid as a reminder that unusual pain or any nonspecific manifestations in pediatric patients, refractory to common treatment should be investigated with extreme diligence not to miss this neoplastic process.

## Background

B- lymphoblastic leukemia/lymphoma (B-ALL/B-LBL) is a group of clonal hematopoietic malignancies committed to the B cell lineage, occurring most commonly in the pediatric age group.^1^ They are characterized by excessive proliferation of leukemic blasts in expense of normal hematopoiesis and patients generally present with leukocytosis or pancytopenia (bone marrow failure). Usual symptomatology include fever, hepatosplenomegaly, lymphadenopathy, bone pain and bleeding.^2^ When the blasts involve the bone marrow and peripheral blood it is termed as B-ALL. However, when the blasts primarily involve the nodal and extranodal sites without significant involvement of the peripheral blood or bone marrow it is termed as B-LBL. Children with B-ALL/B-LBL have good prognosis with overall survival rate of 80% when diagnosed early and treated with modern chemotherapy regimens.^1^ However, atypical presentations of B-ALL/B-LBL may pose significant diagnostic dilemma resulting in delayed therapy.^3^ Although literature describes bone and joint pain in about 40% to 60% of cases of pediatric B-ALL, initial presenting complaint of vaginal pain with normal hemogram is extremely unusual and may distract clinicians from evaluating for a hematopoietic malignancy.^4^

Here, we report a case of a 7-year-old female who presented with symptoms of back, leg and vaginal pain with urinary symptoms and found to have extensive B-LBL involving her spine, lower extremities, pancreas, kidney, brain, axillary lymphadenopathy. There is no hepatosplenomegaly or peripheral blood or bone marrow involvement.

## Case Presentation

A 7-year-old girl with past medical history significant for anxiety and hairline fracture of right humerus 4 months prior, presented with hip, leg, lower back and vaginal pain. Six days prior to presentation, patient started experiencing hip, leg, and lower back pain and 2 days later patient started complaining of vaginal pain. The leg pain did not wake her up at night but gradually worsened she developed weakness with significant limping. The child described “vaginal pain” and reported dysuria and urinary frequency as well as occasionally feeling the need to void but being unable to. There was no pruritus, bleeding or vaginal discharge. Her mother noted dark urine but no gross blood. There were no reported changes in bowel movements though she had a pre-existing history of constipation.

Patient presented to an outside hospital Emergency department (ED) where she was diagnosed with a urinary tract infection (UTI) and started on antibiotics. However, there was no improvement and she returned to ED where she was diagnosed instead with vulvovaginitis and prescribed topical antifungal treatment before she ultimately returned for a third time and was transferred to our tertiary care center for further evaluation of these symptoms. On physical examination, the child was afebrile with mild tachycardia and hypertension. She was not in acute distress but anxious and tearful. She appeared well-developed with normal weight and body mass index². Examination was notable for non-tender right clavicle mass, mild erythema of labia majora and minora and tenderness to palpation in inguinal region but no mass was noted. No cervical or inguinal lymphadenopathy, and no hepatosplenomegaly were appreciated. She had point tenderness on the lumbar and sacral spinous processes, tenderness of hips, thighs, knees, and upper calves bilaterally, decreased active and passive extension of knees bilaterally, full flexion present, full range of motion in bilateral ankles. There was no swelling or deformity. The child lay on her right side with hips and legs flexed and complained of pain when laid on back for examination. Deep tendon reflexes were present but patellar were noted to be slightly diminished bilaterally. Her gait was notable for a shuffling wide based gait. Of note, her mother reported that the right clavicle mass had been evaluated by her orthopedic surgeon, who stated this was resultant from her recent humerus fracture. In addition, mother suspected her symptoms could be due to anxiety as there were recent severe stressors in the family. Four months prior, around the time of the humerus fracture, all family members, except our patient, contracted COVID19 and father of child was severely affected and continued to remain hospitalized up to the time of patient’s presentation to us. The primary pediatric team recognized her back and leg pain as her primary problem and considered rheumatologic process such as juvenile idiopathic arthritis to be most likely versus infection. Her vaginal/urinary complaints were believed to be unrelated and thought to be due to non-fungal vulvovaginitis. Laboratory studies revealed a normal complete blood count (CBC) with elevated erythrocyte sedimentation rate of 87 mm/hour and c-reactive protein (CRP) of 3.9 mg/dl.

**Figure 1.**
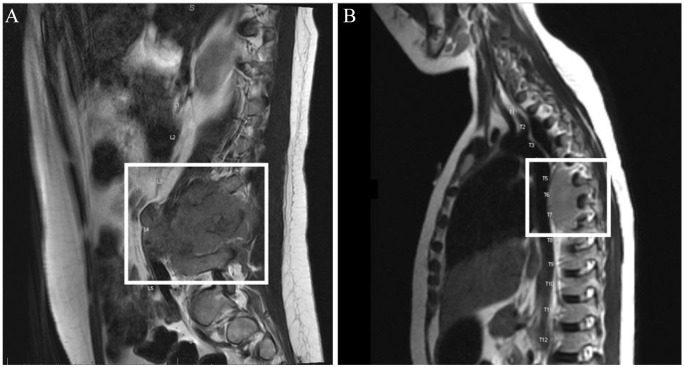
(A) Sagittal pre-contrast T1-weighted fat-saturated magnetic resonance imaging of lumbar spine demonstrates marrow replacing lesion within the L4 vertebral body with extension into the epidural space and associated cortical destruction of the anterior and posterior cortex with bulging of the anterior and posterior longitudinal ligament and (B) epidural extension of tumor extends along the dorsal aspect of the vertebral bodies from the L3 to the L5 level. There is circumferential epidural tumor which extends from the L3-L4 level to the L5 level. There is involvement of the spinous processes as well as the bilateral pedicles.

Magnetic resonance imaging (MRI) of the spine (Figure 1) and lower extremities were performed and showed expansile lesion of the L4 vertebral body with critical epidural compression of the cauda equina and severe spinal canal stenosis at the L4 level. There was also a large mass within and extending from the T6 vertebral body with epidural involvement resulting in severe spinal canal stenosis and suspected compressive myelopathy at the T6 level. There is pathologic compression fracture of the T6 vertebral body with resultant focal kyphosis of the thoracic spine. Additional marrow infiltration of the T4 vertebral body extending to the posterior elements resulting in mild spinal canal narrowing was noted. Multiple marrow lesions were seen involving the bilateral lower extremities, many of which were associated with soft tissue components. Upon completion of MRIs, she noted severe weakness of the bilateral lower extremities with saddle anesthesia. She was unable to urinate and it was noted that there had been no urine output for some time.

This immediately led to emergency surgery for L4 spinal canal decompression and biopsy of the epidural mass. The frozen section showed small round blue cell tumor with probable differential of Ewing’s sarcoma, rhabdomyosarcoma, neuroblastoma, lymphoma, and other small round cell tumors. Alpha-feto protein (AFP), beta human chorionic gonadotrophin (bHCG), vanillylmandelic acid (VMA), and Homovanillinic acid (HVA) were ordered, and all were within normal limits. The child was started on dexamethasone for management of spinal cord edema, while awaiting pathology results.

**Figure 2.**
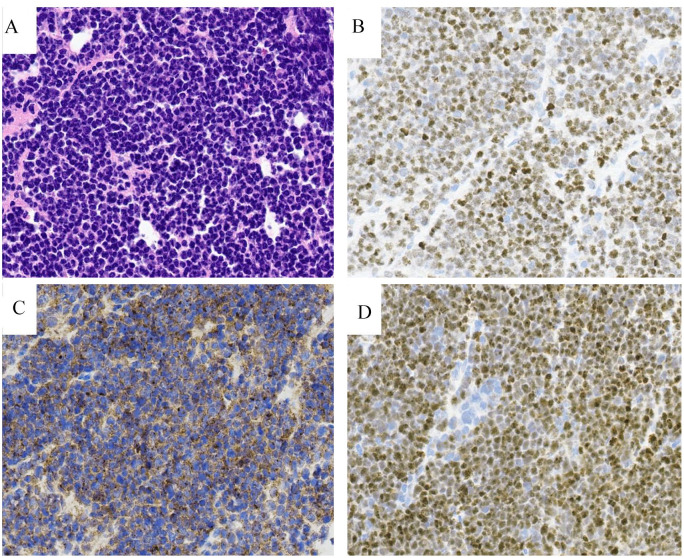
(A) The specimen shows multiple fragments of bone and soft tissue involved by a diffuse atypical lymphoid infiltrate with patchy crush artifact, composed of small to medium sized cells with high N:C ratio, scant cytoplasm, hyperchromatic nuclei, and inconspicuous nucleoli. Apoptosis is present. No necrosis is appreciated. Immunophenotypically, the atypical/neoplastic cells are positive for PAX-5 (B), CD19 (C), and TdT (D). Magnification 400×.

The final pathology of the epidural mass at L4 spine showed (Figure 2A-D) a diffuse atypical lymphoid infiltrate composed of small to medium sized cells with high N:C ratio, scant cytoplasm, hyperchromatic nuclei, and inconspicuous nucleoli. Immunophenotypically, the neoplastic cells are positive for, TdT, CD79a, PAX-5, CD10, CD22, and focal CD19 and negative for CD45, CD20, T cell markers (CD3 and CD5), myeloid markers, melanoma markers, and epithelial markers. Several mesenchymal markers were examined to differentiate round cell tumors, such as Ewing’s sarcoma or rhabdomyosarcoma, but none yielded significant findings. A limited flow cytometry study was performed due to low cellularity and viability (approx. 8%) and did not show any immunophenotypic aberrancies. The findings are compatible with B-lymphoblastic lymphoma (B-LBL). Staging bone marrow biopsy was negative. Cerebrospinal fluid (CSF) was not obtained at diagnosis due to technical challenges given the location of the tumor. Full evaluation including MRI brain, MRI abdomen, and Positron emission tomography-computed tomography (PET-CT) showed extensive disease involving bilateral parietal bone masses with epidural extension/components but without brain invasion, right kidney, and pancreas and bulky right axillary adenopathy with suspected pathologic fracture of the right proximal humerus.

The child was initiated on systemic chemotherapy with vincristine, daunorubicin and Peg-aspargase with excellent toleration. She completed an extended course of Decadron, which was initially prescribed for cauda equina syndrome, and extended as part of her chemotherapy. Her clavicular swelling quickly improved as did the pain in her back and legs. Repeat MRI and PET scan at the end of induction therapy indicatedabout 1 month later showed significant decrease/resolution of the abnormal signal in the right axillary adenopathy, and bone lesions. She was discharged with need for ongoing scheduled urinary catheterization. On the day 22 of consolidation therapy (cyclophosphamide, cytarabine, mercaptopurine, Vincristine, and Peg-aspargase) she started to notice improvement in her bladder symptoms.

## Discussion and Conclusions

Urinary complaints are among the more common reason for presentation to urgent care or ED for young girls. Typically, these are associated with infections (UTI or vulvo-vaginitis) or sexual trauma and very rarely neoplastic processes. Our patient presented in the emergency multiple times with the complaints of urinary symptoms and vaginal pain and was empirically treated with antibiotics and antifungals without symptomatic improvement and dictated the clinical attention to a potential gynecological etiology. However, extensive imaging with biopsy of the lesion revealed B-LBL.

B-ALL is the most common childhood hematologic malignancy characterized by infiltration of leukemic blasts in blood and bone marrow in ALL or in primary nodal/extranodal sites in LBL. Sometimes, there is an overlap between ALL and LBL, and a combined diagnosis is commonly rendered in hard to define cases.^5^

Pediatric patients with ALL/LBL most commonly present with fever, hepatosplenomegaly, lymphadenopathy, bone pain and bleeding.^2^ Musculoskeletal manifestations like back pain, hypercalcemia, fractures, loss of mobility, and deformity are also frequently seen (approximately 40%-60%) in patients with ALL/LBL.^2,4-7^ Unusual presentations of pediatric ALL/LBL include osteoporosis,^8,9^ intussusception,^10^ skin involvement,^11^ and palpable back mass.^12^ However, in most of these cases there is some degree of either bone marrow or blood involvement at the time of presentation.^13–15^

In this case, the unusual complaints of urinary and vaginal pain were due to inadequate bladder voiding and urinary retention causing bladder distention as result of spinal cord compression. This reflects the importance of using objective findings such as urinalysis and physical exam findings, since young children cannot fully describe their symptoms. The child had not had bowel movements for several days, likely another sign of spinal cord compression, but it was believed to be her “normal” constipation. Further, the clavicle swelling was noted by admission team but it was accepted that this was related to her primary humerus fracture as that was suggested by the orthopedic surgeon although that did not really make sense, an example of anchoring bias. This case highlights the importance of recognizing back pain and bone pain in general as very unusual and alarming complaints in young children, unlike in teenagers and adults.

In conclusion, our case describes an unusual presentation of B-LBL and can aid as a reminder that ALL/LBL diagnosis should be considered for all pediatric patients with unexplained bone pain, bone lesions, and other non-specific pain manifestations.
